# The paradox of nest reuse: early breeding benefits reproduction, but nest reuse increases nest predation risk

**DOI:** 10.1007/s00442-019-04436-7

**Published:** 2019-06-17

**Authors:** Andreas Otterbeck, Vidar Selås, Jan Tøttrup Nielsen, Éric Roualet, Andreas Lindén

**Affiliations:** 1 Novia University of Applied Sciences, Raseborgsvägen 9, 10600 Ekenäs, Finland; 2 Department of Ecology and Natural Resource Management, Norwegian University of Life Sciences, P.O. Box 5003, 1432 Ås, Norway; 3 Espedal, 4 9870 Sindal, Denmark; 4 Nedre Hellerudhaugen 5, 1487 Hakadal, Norway

**Keywords:** Anti-predatory strategies, Nest predation, Nest rebuilding, Nest relocation, Breeding phenology, Reproductive investment, Clutch size, Nestling survival

## Abstract

Many animals build new nests every breeding season instead of saving time by reusing old ones. One hypothesis is that nest reuse leads to increased predation risk if predators memorize nest locations and revisit these sites. Here we examine patterns in the prevalence of facultative nest reuse. Further, we relate nest reuse and timing of breeding to nest predation risk, clutch size and nestling survival. We analyse 1570 breeding attempts of the Eurasian sparrowhawk (*Accipiter nisus*) from Denmark (1977–1997) and from two sites in Norway (1985–2017). The probability of reuse varied between study areas, increased in replacement clutches, and was lower in adults compared to 1-year-old breeders. Pairs reusing nests laid their first egg on average 2.6 ± 1.0 SE days later than those building new nests, suggesting they are compensating for an already late breeding schedule. Indeed, reuse increased nest predation risk, but we discovered no other productive effects of reuse. In non-predated nests, late breeders had both smaller clutches and lower nestling survival. We propose that nest predation is a contributing driver to the behaviour of building a new nest each year, whereas nest reuse is a strategy to compensate for delayed onset of breeding, mainly used by inexperienced males.

**Electronic supplementary material:**

The online version of this article (10.1007/s00442-019-04436-7) contains supplementary material, which is available to authorized users.

## Introduction

The nest is a prerequisite for breeding in a wide range of animals. It is particularly relevant for avian species, which use nests for protecting their eggs and nestlings (Hansell 2000). While the nest construction process pose a pre-laying investment, a new nest is usually built annually even if old but structurally intact nests are available for reuse (Lack 1954). The general absence of nest reuse has been surprisingly little studied and its underlying cause has classically been vouched to parasite avoidance (e.g., Clark and Mason 1985). Meanwhile, other hypotheses of the costs and benefits of nest reuse have remained understudied.

A less studied aspect of nest reuse is whether old nests are avoided due to potentially increased risk of nest predation. Nest predation is the primary cause of breeding failure in avian species, posing a strong evolutionary pressure (Martin 1995). An impressive range of counter-measures has been developed by breeding birds (Caro 2005). New-built nests are usually relocated (but within the same breeding site or territory), and its new location is suggested to trick local predators that revisit memorized nests (Sonerud and Fjeld 1987; Sonerud 1985, 1993). However, the scarce evidences for this mechanism are from cavity breeders showing lowered predation risk by nest relocation for both excavators (Nilsson et al. 1991) and non-excavators (Sonerud 1985, 1993; Sorace et al. 2004, but cf. Korpimaki 1987). If the predation risk is acute, this might be a driver of nest relocation. While the generality of these findings is still not thoroughly validated, reuse occur more seldom in species with small body size (Hansell 2000), species breeding in open-cup nests (Erckmann et al. 1990; Redmond et al. 2007) and in the tree canopy (Martin 1995); all known to involve higher predation risk than nest cavities (Lack 1954).

The choice to reuse or build a new nest can be viewed in the framework of animal decision-making; an adaptive trade-off between the costs (risks) and benefits (Lima and Dill 1990). Most strikingly, reuse of existing nests might be advantageous through reduction of time and energy spent pre-laying on nest preparation (Weeks 1978; Conrad and Robertson 1993; Curson et al. 1996). This in turn may allow for earlier breeding onset (Cavitt et al. 1999; Antonov and Antanasova 2003, but see Redmond et al. 2007) or energy reallocated into reproduction (Reid et al. 2000). A few studies show positive productive effects of reuse possibly related to less effort pre-laying (Weeks 1978; Conrad and Robertson 1993; Cavitt et al. 1999), while other studies do not support this (Redmond et al. 2007; Antonov and Antanasova 2003; Jiménez-Franco et al. 2014). Old nests may also provide a cue of past successful breeding, shown by two particular large raptors being prone to reuse existent nests during reoccupancy or new establishment in old territories (Jiménez-Franco et al. 2014). The lack of a consistent pattern might suggest reuse being a facultative choice conditioned on local circumstances and individual state.

It is valuable to study the effects of reuse in an open-nest breeder instead of a cavity breeder, as the latter group is often constrained by lack of available cavities (Newton 1994, 1998; Cockle et al. 2011) and may be biased by nest box experiments (Møller 1989). Small cavity openings as such provides protection against some predators (e.g., Caro 2005), suggesting open-nest breeders may in general be more vulnerable to nest predation. We coin the term facultative nest reuse, to emphasize that individuals may flexibly decide to build or reuse nests based on their current situation. Revealing the variables affecting the decision to reuse nests, is important in order to understand how this widely spread life history trait is regulated.

We use the Eurasian sparrowhawk (*Accipiter nisus*)—hereafter sparrowhawk—as a model species for the cost and benefits of facultative nest reuse. The sparrowhawk is particularly well suited for this study, because the males contribute most to the building of the nest, whereas females add the inner lining of the nest in the final stages (Newton 1986). The nest building is time-consuming: males typically spend around 100 h constructing the nest. The males usually build a new nest annually, but occasionally reuse old ones. Sparrowhawks are partial migrants, and hence, their wintering strategy might affect the time available for nest building (Newton 1986).

We here first describe the spatiotemporal patterns of reuse to draw a general picture of when nests are reused with respect to age of parents, timing of breeding and replacement clutches. Second, we investigate the consequences of nest reuse and the onset of breeding with respect to breeding performance, in terms of nest predation, clutch size, and nestling survival. As the main negative effect, we hypothesize that local predators memorize or can identify old nest locations increasing the predation risk of reused nests (Sonerud 1985; Sonerud and Fjeld 1987). Moreover, we set a two-tailed hypothesis stating that reusing old nests might induce either benefits or costs in the production of young (clutch size and nestling survival). A positive effect of reuse on reproduction could be expected due to less energy spent pre-laying, possibly reallocated into offspring, or as a result of advanced breeding onset. Negative effects could be mediated through parasites or lower quality (e.g., poor insulation) of old nests.

## Materials and methods

### Study species

The sparrowhawk is a small top predator, specialized in catching small birds (Newton 1986). It breeds in open nests located in trees usually situated about 1/3 from the top. In contrast to their larger relative—the Northern goshawk (*Accipiter gentilis*)—sparrowhawks mostly build new relocated nests annually, and do not cover their nests with greenery. However, they occasionally reuse old nests. Since annual relocation within a nesting range will usually be situated within close range of their previous nest (only within some tens of meters), sparrowhawks typically establish core areas with accumulation of old nests (Newton 1986) that could be reused, making it a particularly suitable model species for studying the effects of reuse vs. building new nests. This species also display age-related assortative mating (Newton et al. 1981; Newton 1986).

In the Nordic countries, sparrowhawks mainly nest in Norway spruce (*Picea abies*), Scots pine (*Pinus sylvestris*), and birch (*Betula* spp.). Their preference for nesting in dense forests could be an adaptation to avoid goshawks and pine martens (*Martes martes*) (e.g., Selås 1997)—which are thought to be the most important predators of sparrowhawk nests. Pine martens regularly predate on birds and squirrels (Storch et al. 1990). They are good climbers and can use old sparrowhawk nests as resting places (Sonerud 1985; Newton 1986).

### Study areas

For this study we used data from two study areas in Southern Norway (Oslo and Aust-Agder), and from one larger area (two closely located sub-areas) in Denmark (summarized in Table 1). These data were originally collected for studying different aspects of the species’ breeding biology. Data from Oslo (60°00´N, 10°50´E) were collected by E.R. and A.O. in 2001–2017. Breeding sites in this area were located in both coniferous and deciduous forest ≤ 400 m above sea level, but nests were mainly built in spruce. The nests were situated in dense stands of younger trees (25–40 years) near forest edges and agricultural landscapes. The mean nest height in Oslo was approximately 9 m. The other Norwegian data set was collected by V.S. in Aust-Agder (58°43´N, 8°44´E) in 1985–1999. The area covers about 250 km^2^, and is situated 100–300 m above sea level. The area is hilly, sharply undulating, and dominated by forests, which are a fine-grained mosaic of young, medium-aged and old coniferous, mixed and deciduous stands, with Scots pine, Norway spruce, sessile oak (*Quercus petraea*), aspen (*Populus tremula*) and birch as the dominant tree species. The Danish data were collected in 1977–1997 by J.T.N. in Vendsyssel comprising 2417 km^2^, where the main effort was within two sub-areas, one 68-km^2^ area around Sindal (57°28´N, 10°10´E) and a 436-km^2^ area west of Hjørring (57°28´N, 10°00´E). The first sub-area is open farmland with scattered forests, often connected with hedges. Plantations and forests constitute 16.2% of the area. A total of 95% of plantations and forests are covered with intensively managed conifers. The second sub-area is mainly intensely cultivated farmland with small plantations sized 2–40 ha. Only 1.9% of the area is covered with forest. The two Danish sub-areas are only 8 km from each other, and therefore, they were in our analyses treated as one study area. In contrast to Norway, there are no pine martens in the Danish study area where the main nest predator of sparrowhawk is the goshawk, and the beech marten (*Martes foina*), which is common. Furthermore, the sparrowhawks in the Danish study area are mainly non-migratory (approximately 2% migrates). This contrasts to the situation in Norway, where a large part of the population migrates south for the winter, the overwintering birds mainly being established adults. The Danish sparrowhawks are in many cases prone to initiate a second breeding attempt the same season if the first one fails, whereas the Norwegian pairs usually do not.

**Table 1:** Description of the variables included in the analyses

Variable name	Explanation	Type	Values/levels	Subset	Usage
Breeding status					
Reuse	Breeding in reused nest	Binary factor	“no”, “yes”	All	*y*, *x*
Nest.predated	Nest was predated	Binary factor	“no”, “yes”	All	*y*
Clutch.size.f	Number of eggs in clutch	Ordinal, factor	“2”–”6”	DK	*y*
N.young	Number of young in brood	Numeric	2–6	DK	*y*
Laying.day	First egg, day of year: 1st May = 1	Numeric	(− 15) to − 54	DK	*y*, *x*
Detect.day	Centred day of year nest found	Numeric	(− 25.1) to − 41.9	OS	*x*
Replacement	2nd breeding attempt after failure	Binary factor	“no”, “yes”	DK	*x*
Parental age					
Female.age	Age of breeding female	Binary factor	“2cy”, “adult”	All	*x*
Male.age	Age of breeding male	Binary factor	“2cy”, “adult”	All	*x*
Spatiotemporal variables					
Area	Study area = dataset	Factor, 3 levels	“OS”, “AA”, “DK”	All	*x*
Year.c	Centred year, for temporal trend	Numeric	(− 10.5) to − 9.4	All	*x*
Territory	Territory ID (name)	Factor, 341 levels	Not shown	All	*r*
Year.f	Year ID, for annual variation	Factor, 36 levels	“1977”–“2014”	All	*r*
Year.Area	Interaction of year. f and Area	Factor, 52 levels	Not shown	All	*r*

The variables are divided into three groups, relating to breeding status, parental age, or spatiotemporal environmental variation. Information listed include the variable name used, a brief explanation, the type of variable, the range of values (or factor levels), study areas for which the variable is available (All—all study areas, DK—Denmark, OS—Oslo), and usage of the variables in the analysis (*y*—response variable, *x*—explanatory variable with fixed effects, *r*—random effects on the intercept). In variables related to parental age “2cy” refers to 2. calendar year (1-year-old) birds, while “adult” refers to older age classes. The quantitative variable “Year.c” was centred to zero mean for each data set separately. “Year.f” spans the time period 1977–2014 but lacks data from 2001 to 2002

### Description of the data

In our analyses of facultative nest reuse, we only used data from breeding events where both breeding success—including occurrence of nest predation—and nest quality (reused or new) were known. Nests are flattened by use, and hence reused nests could be visually identified in field. However, reuse requires repairs, which makes identification a bit more difficult. Detailed knowledge on the location of old nests eliminated the problem, although nests falling down during winter pose challenges to keeping exact track. New nests are usually relocated 10–40 m away from previous ones. To avoid errors in assessing nest quality, we disregarded all breeding attempts from the year when a territory was discovered (or re-discovered after many years of no activity). We also removed breeding attempts that did not lead to laying a clutch of at least two eggs, and cases where breeding was interrupted, e.g., because one of the parents were preyed upon. This compiled a dataset with a total of 1570 breeding attempts: 29 attempts in 14 different territories in Oslo, 411 breeding attempts in 94 territories in Aust-Agder, and 1130 breeding attempts in 233 territories in Denmark.

We define nest predation as instances where either eggs or nestlings were predated at the time they were still tied to the nest. When a breeding pair failed during the season, the cause was investigated by examining the ground around the nest tree for cues (e.g., broken egg shells), and by examining the nests when possible. In the Danish dataset we had cases where eggs were known to be predated by Eurasian jay (*Garrulus glandarius*), which may indicate that the breeding attempt has been aborted earlier, leading to unprotected eggs vulnerable to predation. Therefore, we removed such events if no further information was available. In Aust-Agder and Denmark, several breeding attempts were assumed aborted by human interference either by nest-looting or obvious disturbances early in the season, and these cases were also excluded from our dataset.

Clutch size was collected by visual inspection of the nests, often multiple times during a single breeding season, except in the Danish study area where clutch size was determined during ringing of the nestlings at the age of 2–3 weeks. The number of survived nestlings were estimated at fledging, by combining visual observation shortly before and immediately after fledging, when being fed by the parents, usually still close to the nest tree. As a measure for the onset of breeding, laying date of the first egg was back-calculated based on age determination of the nestlings (Newton 1986).

### Variables and statistical analyses

The variables analyses are summarized in Table 1. The data collected at the three study areas were somewhat heterogeneous, implying that all variables studied were not recorded by all authors (Table 1). To keep the sample sizes and statistical power sufficiently high, we occasionally used different subsets of the full dataset in different analyses (subsets reported in Table 2). Laying date of the first egg (continuous variable “Laying.day”) was coded with a running number, such that 1st May is 1 (2nd May is 2, 30th April is 0, 29th April is − 1). The numeric variable “Year.c” is study year centralized (to zero mean) for each data set separately, describing the average temporal trend within datasets. The factor variable “Area” will hence capture varying levels between the data sets from different study areas, i.e., spatial effects plus possible effects of differing study periods. We treated all explanatory binary variables as numeric (starting point 0 and 1), and centralized them to zero mean, separately for each subset of data. This approach provides identical estimated effects as if modelled as a factor variable, but ensures that the model intercept describes the average situation in the focal subset of data (Schielzeth 2010).

**Table 2:** Summary of the (generalized) linear mixed models (#1a–#5) used in this manuscript, with reference to the variables described in Table 1

Model	Response	Fixed	Random	Model type	Subset	*n*
Reuse level and spatiotemporal variation						
#1a	Reuse	Area + Female.age	Territory + Year.f + Year.Area	Binomial	All	1334
#1b	Reuse	Area + Male.age	Territory + Year.f + Year.Area	Binomial	All	469
#1c	Reuse	Replacement	Territory + Year.f	Binomial	DK	1129
#2	Laying.day	Reuse + Replacement + Year.c	Territory + Year.f	Gaussian	DK	902
Consequences by nest reuse						
#3a	Nest.predated	0 + Area + Area:Reuse + Year.c	Territory + Year.f + Year.Area	Binomial	All	1257
#3b	Nest.predated	Reuse + Detect.day	Territory + Year.f	Binomial	OS	29
#4	Clutch.size.f	Reuse + Replacement + Laying.day	Territory + Year.f	Ordinal	DK	736
#5	N.young/Clutch.size	Reuse + Replacement + Laying.day	Territory + Year.f	Binomial	DK	736

The model structures are described in terms of the response variables used (Response), explanatory variables with fixed effect (Fixed) and random effects on the intercept (Random), type of model (Model type; Binomial—logit link, binomial error; Gaussian—identity link, Gaussian error; Ordinal—logit link, multinomial error), the subset of data used (Subset; All—all data, DK—Denmark, OS—Oslo) and the total sample size in the analysis (*n*). The models are divided into those describing patterns of reuse and timing of breeding (models #1a–#2), and those investigating the consequences to breeding performance by reuse (models #3a–#5). We centralized all numeric and binary explanatory variables (in column fixed) prior to the analyses

Many of the breeding attempts were from the same territories in different years. To account for further spatial variation and the dependence between data points from the same territory, we included the factor variable “Territory” as a random effect on the intercept in all our statistical models. Likewise, we included study year as a factor variable “Year. f” with random effects in all models. If several areas were included, we also applied a random effect of the interaction of year and area (“Year.Area”), to model the area-specific annual variation (“Year.f” taking care of the spatially synchronous share of the variation). There is likely to be such annual variation in breeding variables caused by weather; e.g., cold and rainy conditions during early breeding season are known to negatively affect breeding success (Newton 1986).

All statistical analyses were done in R, version 3.4.1 (R Core Team 2017). We fitted linear mixed effects models (LMM) and generalized linear mixed models (GLMM), using the package “lme4” (Bates et al. 2015). For the LMMs we applied the Satterthwaite approximation for degrees of freedom using package “lmerTest” (Kuznetsova et al. 2015) in order to get reliable estimates of statistical significance. For analysing clutch size we applied an ordinal mixed model, using the package “ordinal” (Christensen 2018). All statistical tests presented are two-tailed, with *α* = 0.05.

### Modelling patterns of facultative nest reuse

To study the probability of nest reuse, we applied three different logistic models (GLMMs with logit link and binomial error distribution), all with “Reuse” as the binary response variable with two factor levels: “no” (new nest) and “yes” (old nest) (Table 2). In the two first models (#1a and #1b), we studied how reuse might depend on female and male ages (factors “Female.age” and “Male.age”, respectively) when accounting for “Area”. These were investigated in separate models to keep sample size sufficiently high, as the age of both parents was known in a fairly small fraction of the breeding attempts. In model (#1c), we investigated whether reuse of nests is more likely during replacement clutches (factor “Replacement”). In this particular analysis, we used data only from Denmark where long seasons allow replacement clutches; this is rare further north (e.g., in Norway).

To assess how facultative nest reuse affects the timing of breeding (#2), we set “Laying.day” as the response variable in a linear mixed model (with Gaussian error distribution). “Reuse”, “Replacement” and “Year.c” were the explanatory variables, again only for the Danish data set (Table 2).

### Models for nest predation

To investigate whether occurrence of nest predation was higher in breeding attempts reusing old nests, we applied logistic GLMMs (logit link and binomial error), where “Nest.predated” was the response variable (Table 2). Model (#3a) accounted for different general levels of predation and effects of reuse at the different study areas (separate intercepts and slopes; interaction between “Area” and “Reuse”). We also estimated a common temporal trend within the datasets (“Year.c”). Again, “Territory”, “Year.f” and “Year.Area” were random effects accounting for unexplained spatiotemporal variation caused by the environment.

Possible predation events are obviously more likely to be identified in the period after an active breeding attempt has been detected. Therefore, breeding attempts in new-built (previously unknown) nests, predated early in the season, may go undetected and bias the results towards more predation events among old nests. In the Danish dataset, we did not include predation events where Eurasian jay was identified as the predator, because jays are likely to predate eggs after the breeding attempt has, for some reason, been aborted. In the Oslo dataset, where territory activity was particularly regularly monitored throughout the breeding season, we wanted to minimize this source of bias by removing all breeding events detected later than day of year 148, which marks the initiation of the hatching period in Oslo. We also removed breeding events where the last visit was earlier than the average time point for fledging (day of year 186), when nest predation ceases. Finally, to validate our qualitative results from model #3a, we fitted model #3b, for the Oslo dataset only, where we explained nest predation with reuse and the day of detection of the active nest (“Detect.day”) as a covariate, approximating the available time span for effective detection of predation.

### Models of clutch size and nestling survival

Apart from nest predation, we studied other effects on productivity by examining how clutch size and survival of nestlings were affected by reuse, clutch replacement and timing of breeding in non-predated nests. For these analyses we excluded all breeding attempts where the exact number of eggs was not known (only a minimum clutch size). We also excluded cases where no nestlings survived, as these are typically associated with some kind of interrupted breeding (e.g., nest destroyed or predated, or parents predated).

We studied clutch size using an ordinal mixed model (#4), or cumulative link model, which uses a logit link for modelling variation in the probability of reaching the next ordinal level. Clutch size was treated as an ordinal response variable with five levels: “2 eggs”, “3 eggs”, “4 eggs”, “5 eggs”, and “at least 6 eggs”, while the explanatory variables were reuse, replacement clutch and laying date (Table 2). Naturally, timing of breeding and replacement clutches correlate strongly, but both may possibly contribute with unique effects.

For studying survival of nestlings in any given clutch, we used a logistic GLMM (#5), with “N.young” (successes) and “Clutch.size”–“N.young” (failures) as the binomial response variable(s). Again, the explanatory variables were reuse, replacement clutch and laying date (Table 2).

## Results

### Facultative nest reuse

**Fig. 1:**
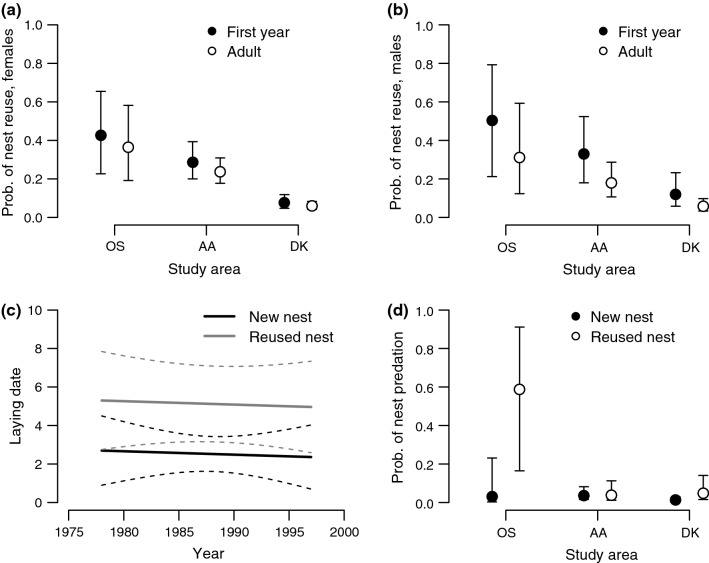
Aspects of nest reuse and nest predation in Eurasian sparrowhawk (*Accipiter nisus*). **a** The prevalence or reuse varies between study areas, but is not significantly affected by female age. **b** However, first-year males were more likely to reuse old nests compared to older individuals (the effect of age is common for all areas on the logit-scale; back-transformed to probabilities in the figure). **c** Laying of the first egg occurred ca 2.6 days later in reused nests, the temporal trend being non-significant. **d** When old nests were reused, the risk of nest predation increased compared to when new relocated nests were built. The effects were statistically significant in Oslo (OS) and Denmark (DK), but not in Aust-Agder (AA). All whiskers and dashed lines represent 95% confidence intervals around the fitted average

The level of nest reuse varied between the datasets (Wald test: *W* = 24.6, *df* = 2, *p* < 0.001; Fig. 1a, b), being much lower in Denmark than in Oslo (model #1a: − 2.21 ± 0.46 SE), while the level in Aust-Agder did not differ significantly from Oslo (ESM Table S1). Female age had no significant effect on reuse prevalence (Fig. 1a), but adult males reused nests significantly less often compared to 2 cy males (model #1b: − 0.81 ± 0.37 SE, *z* = − 2.16, *p* = 0.031; Fig. 1b).

Pairs where the male and female showed age-related assortative mating with matching ages (2cy–2cy, adult–adult) were clearly overrepresented in the dataset (Pearson’s χ^2^-test with Yates’ continuity correction: *χ*^2^ = 95.3, *df* = 1, *n* = 464, *p* < 0.001). The observed proportion of matching age was 85.8% while the expected proportion under the null hypothesis of independence is 73.7%.

In Denmark, the probability of nest reuse increased significantly in replacement clutches (25 instances) within the same season (model #1c: 2.33 ± 0.55 SE, *z* = 4.27, *p* < 0.001). Reused nests showed a delayed laying date of 2.60 days ± 0.96 SE (model #2: *t* = 2.71, *df* = 879.0, *p* = 0.007) (Fig. 1c). We found no statistically significant temporal trend (within data sets) in laying date. All estimated parameters of the models related to reuse are presented in ESM Table S1.

### Predation risk in relation to nest reuse

The level of nest predation in new-built nests varied between the three study areas (model #3a; Wald test: *W* = 6.16, *df* = 2, *p* = 0.046; Fig. 1d). Also the effect of nest reuse on predation risk varied between study areas (Wald test: *W* = 7.11, *df* = 2, *p* = 0.029; Fig. 1d; ESM Table S2), being largest in Oslo (3.80 ± 1.40 SE, *z* = 2.71, *p* = 0.007), still clear in Denmark (1.32 ± 0.57 SE, *z *= 2.32*, p* = 0.020), but non-significant in Aust-Agder (− 0.051 ± 0.570 SE, *z *= 0.089, *p* = 0.929) (Fig. 1d). In the validation analysis, based on the Oslo dataset, the effect of reuse on predation was still clear (model #3b: 3.95 ± 1.43 SE, *z* = 2.76, *p* = 0.006), and we found no relationship between the timing of nest detection and the predation level (− 0.056 ± 0.044 SE, *z* = − 1.26, *p* = 0.206). No temporal trend in nest predation rate was observed within the data sets. All estimated parameters of the models on predation risk are given in ESM Table S2.

### Clutch size and nestling survival in non-predated clutches

**Fig. 2:**
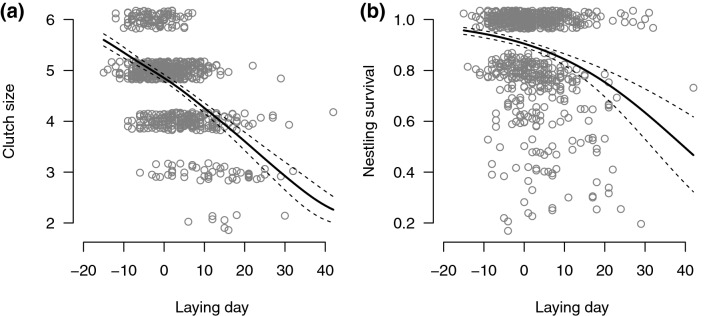
There were statistically significant negative effects of laying day on **a** clutch size and **b** survival of nestlings (i.e., proportion of eggs hatched and surviving until fledging). The partial effects of laying day are illustrated as the predicted clutch size and nestling survival from generalized linear mixed models (models #4 and #5, respectively), where the other explanatory variables are set to their average values. The grey open circles are data points with jitter added on the y-axis to facilitate illustration. The black lines are the model fit, while the dashed lines are 95% confidence limits for the model fit. In model #4 the confidence limits are obtained with a parametric bootstrap procedure with 2000 repetitions

Laying date of the first egg had a highly significant negative effect on clutch size (model #4: − 0.178 ± 0.014 SE, *t *= − 12.8, *n *= 736, *p* < 0.001). Translating the ordinal model partial effect of laying day to predicted absolute clutch size, results in an approximately linear relationship corresponding to one egg less when laying is delayed by 17 days (Fig. 2a). Neither reuse, nor replacement clutch had any additional effect on clutch size.

Similarly to clutch size, nestling survival clearly decreased with laying day (model #5: − 0.057 ± 0.008 SE, *z* = − 7.17, *n* = 736, *p* < 0.001; Fig. 2b). Reuse had no effect on nestling survival, but nestlings of replacement clutches had much better survival than expected from their late timing (2.75 ± 1.06 SE, *z* = 2.60, *p* = 0.009). All parameter estimates for models #4 and #5 are given in ESM Table S2.

## Discussion

### Facultative nest reuse

We found that the level of nest reuse varied across our study areas. While there was no statistical difference between Oslo and Aust-Agder, the Danish study area stood out by having a significantly lower nest reuse rate (Fig. 1a, b). Birds in the Danish population migrate less frequently and the average pair may hence have less time constraints in the beginning of breeding. Within these populations, we identified that nest reuse was particularly common in replacement breeding attempts.

Adult males reused nests less frequently compared to first-year breeders (Fig. 1b). This result provides a novel connection between facultative nest reuse and individual experience (cf. Redmond et al. 2007), i.e., at least the most frequent reusers have no prior breeding experience. Sparrowhawks are partially migratory in the Nordic countries and proportionally more juveniles migrate compared to adults (Newton 1986). Hence, young individuals may be overrepresented within the late arriving fraction of the population, with limited time for nest construction. Obviously, nest reuse by first-year breeders must exclusively concern other individuals’ old nests. Male sparrowhawks have the sole responsibility of building the nest (Newton 1986), which may explain the non-significant effect of female age, despite the high level of age-related assortative mating in sparrowhawks (Newton et al. 1981), in our case approximately 85%. Our results reassure our view that nest reuse pose an individual facultative decision, varying across and within populations, and even changing with age.

Reuse of nests (during first attempts) entailed delayed laying compared to new nests (Fig. 1c). While this may seem counterintuitive, reuse can still be applied to save time pre-laying by pairs that are delayed in their schedule, despite the higher risk for nest predation. Although the laying date of the first egg in reused nests was 2.6 days later than in new nests, laying would likely have occurred considerably later if these pairs would have had to build a new nest. Apparently, the time saved does not fully compensate for the late schedule. Furthermore, reuse was more common in replacement clutches (when first attempts failed), which were laid on average 4 weeks later, compared to regular first clutches, possibly being the only remaining option to reproduce that season. In contrast to this finding, the multi-brooded Eurasian blackbird (*Turdus merula*) have been shown to avoid reusing the same nest after an unsuccessful attempt (Wysocki 2004), likely because it has more time to re-breed and invests less time and energy in building its nest.

### Predation risk in relation to nest reuse

The predation level in new nests showed variation across the three areas included in our study, but no temporal trend (Fig. 1d). The average predation level of approximately 4.4% in our study was considerably higher than the level reported earlier (2%) for sparrowhawk in Great Britain (Newton 1986) possibly reflecting that neither of the predators pine marten or goshawk occurred in the British study area.

As hypothesized, we found that nest predation risk was higher for reused nests than for new nests, but this effect was only significant in 2 of the 3 study areas. This finding embraces nest relocation as part of an anti-predatory strategy, possibly reflecting that predators (e.g., martens) may re-check memorized nests (Sonerud 1985; Sonerud and Fjeld 1987; Sorace et al. 2004). The beneficial effect of nest relocation has earlier been shown to be greatest in nests predated in the past such as for the cavity breeding Tengmalm’s owl (Sonerud 1985, but cf. Styrsky 2005), and such nests seem to be avoided (Harvey et al. 1979; Dow and Fredga 1983). Additionally, Wysocki (2004) found that within-season nest reuse in Eurasian blackbirds occurred more frequently in nests with successful breeding that were high above the ground and well concealed, further illustrating that predation is a key concern during nest site selection. Another factor relevant to predation risk is nest tree height (e.g., McIvor and Healy 2017), but we did not have sufficient data to examine this.

In our study, the mechanism behind the increased predation risk in reused nests should be interpreted with some care. Instead of memorizing old nest locations, predators may use other cues from old breeding events. For instance, martens may more easily find breeding attempts in old nests if pre-existing olfactory cues are accumulated after more than one breeding season. It is also possible that the young are more vocal in reused nests due to higher parasite burden (Christie et al. 1996), which in turn facilitates predation (Leech and Leonard 1997).

The association between nest reuse and elevated predation risk might explain why species routinely reusing nests tend to be large raptors (e.g., Saga and Selås 2012; Jiménez-Franco et al. 2014), which invest in large robust nest constructions and have fewer natural enemies. For example, the goshawk, which is a close relative to the sparrowhawk, frequently reuses old nests. It has a much larger body size and is likely to repel many nest predators, such as martens. The rise in predation risk by reuse differed among our three spatially separate study areas. Birds have been shown able to assess the prevailing predation risk, and to adjust their reproductive strategies accordingly (Lima and Dill 1990; Fontaine and Martin 2006). In our study, both the level of reuse and the effect of reused nests on predation risk were particularly high in Oslo, though with a noticeable uncertainty due to small sample size from this particular study area. Assuming that reuse is a facultative decision with risks reflecting local predation levels, reuse should be particularly avoided in areas with heavy nest predation. Our study, with only three areas investigated, is not sufficient for answering that question. However, future studies with spatially extensive data may test whether the level of nest reuse tracks local predation pressures, and whether its risks are compensated by other anti-predatory countermeasures.

### Clutch size and nestling survival in non-predated clutches

Laying date typically shows a negative correlation with breeding success (e.g., Perrins 1970; Rowe et al. 1994) so that late breeders experience lowered productivity. Accordingly, we found a strong reduction in both clutch size and survival of nestlings with later timing of breeding (Fig. 2a, b), clearly highlighting the importance of keeping the breeding schedule in sparrowhawks. Replacement clutches showed no additional effect on clutch size, when timing of breeding (laying date) was accounted for. Hence, the typically small replacement clutches could be fully explained by the seasonal decline. Somewhat surprisingly, replacement clutches showed better nestling survival compared to first clutches, considering that the replacement clutches were typically laid very late (predicting lower survival). This could possibly be explained by lower competition between fewer siblings, which enhances individual survival. Additionally, the reduction in survival may not decline throughout the season as steeply as expected by the logistic function (see Fig. 2b), leading to a positive partial effect of replacement clutch in the model.

Perceived predation risk may cause stress and thus induce indirect effects on productivity (Martin 2011). However, our results suggest that presumed increased predation risk by reuse inflicts no additional reduction of the productivity in non-predated nests.

While predation may be a widespread driver of nest relocation across taxa, there is obvious species-specific variation in anti-predator strategies, and likely also in the reasons for nest relocation. For instance, building new nests is typical also in some bird species where predation is not likely to be the driving force for this behaviour. The magpie (*Pica pica*), with approximately the same weight as a sparrowhawk hen, avoids using old nests (Antonov and Antanasova 2003). However, magpie nests are regularly reused by some raptors although being very conspicuous and thus easy to detect for predators (Zhou et al. 2009).

The possible role of parasites in nest reuse aversion yet requires further study. Recent research from another small (but cavity breeding) raptor, the lesser kestrel (*Falco naumanni*), showed that old “dirty” nest boxes was clearly preferred over clean and empty ones (Podofillini et al. 2018), suggesting the opposite patterns than expected from parasite-driven aversion of reuse. Still most relevant studies on reuse and parasites stem from closed nest boxes where clutch size is known to correlate with nest cavity size (Møller 1989), distorting detection of possible negative productive effects. Drawn directly from our results, we suggest that predation risk in sparrowhawks is likely to be a contributing driver for avoiding reuse of old nests.

## Conclusions

We have shown that under normal circumstances, nest reuse is mostly used by inexperienced males and pairs that are delayed for other reasons in order to complete their breeding attempt. We show that first-year males reused nests more frequently than older males. Nest reuse was associated with increased predation risk and slightly later than average laying of the first egg. Both clutch size and nestling survival declined with later laying, highlighting the importance of early breeding. In the future, we suggest that studies incorporate several populations from different areas to reveal whether this anti-predatory behaviour might be adapted to local predation levels.

## Electronic supplementary material

Below is the link to the electronic supplementary material.

Supplementary material 1 (DOC 34 kb)
