# Supplementary material for: The paradox of nest reuse: early breeding benefits reproduction, but nest reuse increases nest predation risk
Source: Oecologia. 2019 Jun 17;190(3):559–68. doi: 10.1007/s00442-019-04436-7 (PMC6647522; doi:10.1007/s00442-019-04436-7)
Supplement: Supplementary file 1 — Supplementary material 1 (DOC 34 kb) [file 442_2019_4436_MOESM1_ESM.doc]

**ELECTRONIC SUPPLEMENTARY MATERIAL (ESM)**

***OECOLOGIA***

**The paradox of nest reuse: early breeding benefits reproduction, but nest reuse increases nest predation risk**

Andreas Otterbeck^1^, Vidar Selås^2^, Jan Tøttrup Nielsen^3^, Éric Roualet^4^ & Andreas Lindén^1^

^1^ Novia University of Applied Sciences, Raseborgsvägen 9. FI-10600, Ekenäs, Finland

e-mail: andreas.otterbeck@novia.fi

^2^ Department of Ecology and Natural Resource Management, Norwegian University of Life Sciences, P.O. Box 5003, NO-1432 Ås, Norway

^3^ Espedal 4 9870 Sindal, Denmark.

^4^ Nedre Hellerudhaugen 5, NO-1487 Hakadal, Norway

*Correspondence: Andreas Otterbeck, Novia University of Applied Sciences, Raseborgsvägen 9. FI-10600, Ekenäs, Finland

E-mail: *andreas.otterbeck@novia.fi*

**Table S1** Parameters for the models on nest reuse and timing of breeding. Models #1a–#1c have the factor variable “Reuse” as the response, while model #2 has the continuous variable “Laying.day” as the response. Fixed effect parameter estimates are reported together with their standard errors (SE), Satterthwaite degrees of freedom (df; if applicable), test statistics (*z-* for models #1a–#1c; *t* for model #2) and *p*-values. For factor variables modelled as contrasts, the relevant factor level is given in parenthesis. For random effects we present the estimated standard deviations.

Model Response Parameter Estimate SE df *z* / *t* *p*

#1a Reuse Intercept –0.512 0.448 – –1.142 0.254

Area (AA) –0.615 0.470 – –1.310 0.190

Area (DK) –2.211 0.463 – –4.777 < 0.001

Female.age –0.261 0.222 – –1.175 0.240

Territory SD 0.046 – – – –

Year.f SD 0.074 – – – –

Year.Area SD 0.367 – – – –

#1b Reuse Intercept –0.681 0.594 – –1.145 0.252 Area (AA) –0.726 0.661 – –1.098 0.272 Area (DK) –2.006 0.650 – –3.087 0.002 Male.age –0.808 0.374 – –2.162 0.031

Territory SD 0.000 – – – –

Year.f SD 0.000 – – – –

Year.Area SD 0.547 – – – –

#1c Reuse Intercept –2.996 0.280 – –10.71 < 0.001

Replacement 2.326 0.545 – 4.266 < 0.001

Territory SD 0.244 – – – –

Year.f SD 0.660 – – – –

#2 Laying day Intercept 2.694 0.459 19.50 5.872 < 0.001

Reuse 2.601 0.958 879.0 2.714 0.007

Replacement 26.99 1.660 868.5 16.26 < 0.001

Year.c –0.018 0.079 18.39 –0.223 0.826

Territory SD 1.983 – – – –

Year.f SD 1.532 – – – –

**Table S2** Parameters for models #3a–#5, on the effects of reuse, which are related to breeding success (nest predation risk, clutch size and fledgling survival). Fixed effect parameter estimates are reported together with their standard errors (SE), test statistics (*z)* and *p*-values. For factor variables modelled as contrasts, the relevant factor level is given in parenthesis. For random effects we present the estimated standard deviations.

Model Response Parameter Estimate SE *z* *p*

#3a Nest.predated Area (OS) –3.055 1.049 –2.912 0.004

Area (AA) –3.275 0.427 –7.662 < 0.001

Area (DK) –4.165 0.396 –10.524 < 0.001

Year.c 0.034 0.033 1.039 0.299

Area (OS):Reuse (yes) 3.804 1.402 2.714 0.007

Area (AA):Reuse (yes) 0.051 0.570 0.089 0.929

Area (DK):Reuse (yes) 1.324 0.571 2.318 0.020

Territory SD 1.365 – – –

Year.f SD 0.000 – – –

Year.Area SD 0.000 – – –

#3b Nest.predated Intercept –1.890   0.749 –2.524 0.012

Reuse (yes) 3.954 1.432 ­­2.761 0.006

Detect.day –0.056 0.044 –1.264 0.206

Territory SD 0.000 – – ̶

Year.f SD 0.000 – – ̶

#4 Clutch size Threshold coef. (2|3) –6.300 0.435 –14.49 –

Threshold coef. (3|4) –3.878 0.232 –16.69 –

Threshold coef. (4|5) –1.219 0.137 –8.925 –

Threshold coef. (5|6) 2.181 0.161 13.58 –

Reuse (yes) 0.059 0.330 0.179 0.858

Laying.day –0.178 0.014 –12.80 < 0.001

Replacement 0.605 0.859 0.704 0.481

Territory SD 0.861 – – –

Year.f SD 0.210 – – –

#5 Survival of nestlings Intercept 2.245 0.079 28.656 < 0.001

Reuse (yes) 0.215 0.253 0.846 0.397

Laying.day –0.057 0.008 –7.173 < 0.001

Replacement 2.753 1.058 2.601 0.009

Territory SD 0.485 – – –

Year.f SD 0.000 – – –
